# Tuberculosis rates in migrants in low-incidence European countries, according to country of origin, reporting country and recency of immigration, 2014 to 2020

**DOI:** 10.2807/1560-7917.ES.2025.30.11.2400489

**Published:** 2025-03-20

**Authors:** Teresa Domaszewska, Anders Koch, Sarah Jackson, Brit Häcker, Jerker Jonsson, Kristina Langholz Kristensen, Hanna Soini, Wouter Arrazola de Oñate, Jean-Paul Guthmann, Barbara Hauer, Mary O´Meara, Karine Nordstrand, Vinciane Sizaire, Gerard de Vries

**Affiliations:** 1 Robert Koch Institute, Berlin, Germany; 2 Statens Serum Institut, Copenhagen, Denmark; 3 Rigshospitalet University Hospital, Copenhagen, Denmark; 4 Health Protection Surveillance Centre, Dublin, Ireland; 5 German Central Committee against Tuberculosis (DZK), Berlin, Germany; 6 Folkhälsomyndigheten/Public Health Agency of Sweden, Solna, Sweden; 7 Nordsjællands Hospital, Hillerød, Denmark; 8 Finnish Institute for Health and Welfare, Helsinki, Finland; 9 BELTA - Belgian Lung and Tuberculosis Association, Brussels, Belgium; 10 Santé publique France, Saint-Maurice, France; 11 National Health Protection Service, Dublin, Ireland; 12 Norwegian Institute of Public Health, Oslo, Norway; 13 FARES - Fonds des Affections Respiratoires, Brussels, Belgium; 14 National Institute for Public Health and the Environment (RIVM), Bilthoven, Netherlands

**Keywords:** tuberculosis, epidemiology, refugees, migrants, screening, incidence rates

## Abstract

**Background:**

As tuberculosis (TB) incidence rates decrease faster in native than migrant populations in European countries, addressing migrant health becomes increasingly important in TB programmes.

**Aim:**

To inform European TB prevention and control policies, we analysed data on TB in migrants in low TB-incidence European countries (TB incidence < 10/100,000 population) during 2014–2020 by migrant origin, destination, and recent vs non-recent immigration.

**Methods:**

Data on migrant TB patients were derived from the European Surveillance System (TESSy) and data on migrant populations from Eurostat or national statistical offices. We calculated annual migrant TB crude incidence rates (CIRs) per country of origin, destination country and year, for all migrants with TB and recently arrived migrants with TB, the latter defined by TB diagnosis within 1 year after arrival in the destination country.

**Results:**

In 2014–2020, 104,371 migrants with TB were reported to TESSy by 20 destination countries. Average annual migrant CIRs were highest in the United Kingdom (43/100,000). Origin countries of most migrant TB patients were India (n = 9,561), Romania (n = 8,345), and Pakistan (n = 7,300). The highest CIRs were found among migrants from Eritrea (480/100,000), Somalia (414/100,000) and The Gambia (343/100,000), and were higher than estimated World Health Organization incidences for those countries. The CIRs among recently arrived migrants appeared higher than in the overall migrant population.

**Conclusions:**

We found substantially higher CIRs in certain migrant subpopulations than others. TB rates in recent migrants appeared to be up to 11 times higher than in corresponding origin countries. Tailored and regularly adapted TB prevention and control strategies are needed.

**Key public health message**

**What did you want to address in this study and why?**

We investigated tuberculosis (TB) crude incidence rates (CIRs) in migrants within low TB incidence European countries in 2014–2020, stratified by country of origin, destination, reporting year, separately for recent and all migrants. The goal was to identify migrants at highest risk of TB and provide information necessary to inform health strategies directed at this key population for TB screening, prevention and care in Europe.

**What have we learnt from this study?**

The TB risk in migrants in Europe depends on their origin and destination, as both migrants from different origin and migrants from the same origin presented varying CIRs depending on destination country. The TB CIRs among migrants differed from the incidence in their origin countries. Also, substantially higher CIRs were found in migrants who arrived at their destination < 1 year before diagnosis compared with all migrants from a given origin.

**What are the implications of your findings for public health?**

The migrant subpopulations with highest TB CIRs, particularly migrants originating from high incidence countries and recent migrants, require targeted, people-centred access to TB prevention, diagnosis and care. The health strategies, including screening for TB, need to be regularly adapted to the changing migration situation.

## Introduction

Tuberculosis (TB) remains one of the leading infectious causes for illness and death worldwide with the highest burden of disease in Asian and Sub-Saharan countries [1]. While the overall incidence of TB decreased in 2000–2023 in countries within the European Union and European Economic Area (EU/EEA), the proportion of TB patients notified in migrants increased [2,3]. In 2022, 33% of all notified TB patients in EU/EEA were diagnosed among migrants [3].

For the northern and western EU countries, the EU countries, through which migrants from Africa and Asia enter the EU [3], and for the United Kingdom (UK), this proportion was substantially higher.

Migrants comprised over 75% of TB patients in Cyprus, Iceland, Malta, Luxembourg, the Netherlands, Norway, Sweden and the UK [3], and the risk of TB in migrants was in certain countries 10 to 20 times higher than among the native populations [4,5]. Thus, migrants are one of the key populations to reach by TB control activities in Europe [6].

Among migrants, TB mostly results from reactivation of TB infection (TBI) acquired in the country of origin or during migration [7,8]. Migrants from countries with high TB incidence (above 100/100,000 population according to the World Health Organization (WHO) [9], further referred to as ‘high-incidence countries’) may have an increased risk due to exposure in the country of origin. Also factors related to circumstances and routes of migration such as overcrowding in camps, poor access to healthcare, stress and malnutrition contribute to the risk [10,11]. The risk of developing TB is highest within 5 years after arrival in the destination country and remains elevated for over a decade following migration [12,13].

Activities to control TB in migrant populations, such as screening for TB disease and/or TBI as part of TB programmes, vary widely in EU/EEA countries[14]. The selection of migrants eligible for screening is usually based either on WHO estimates of TB incidence in the origin countries, applying varying thresholds, or on incidences in specific migrant groups such as asylum seekers [15]. Few national screening policies consider other factors such as actual yields of national TB screening programmes, migration route and potential risk factors during migration and reevaluate these factors [16,17].

While the screening strategies mainly target asylum seekers or refugees, it has been estimated that only ca 11% of residence permits in the EU are issued for asylum reasons [18]. Hence, most migrants residing in the EU are non-EU citizens other than asylum seekers or refugees, as well as migrants from within the EU/EEA. Therefore, the term ‘migrant’ describes a very diverse group, with different assumed risks of TB [2,19].

A study published in 2023 calculated TB incidence rates for migrants from certain countries, some being substantially lower and others substantially higher than WHO estimates for their country of origin [20]. The highest TB incidence rates in Europe were found in migrants born in Afghanistan, Eritrea and Somalia, and varied between the destination countries: for example, in Sweden the TB incidence among Somalian-born migrants was over 10 times lower than in Italy [20].

To inform policies and develop harmonised screening strategies for those most at risk, a better understanding of TB risk in migrants according to origin and over time since migration is needed. The aim of this study was to investigate the TB risk among migrants in low-incidence European countries  (TB incidence < 10/100,000 population per year) between 2014 and 2020, according to country of origin, destination country and time after arrival by calculating migrant crude incidence rates (CIRs) using official data on TB notifications and population data.

## Methods

### Definitions

We used the term ‘migrants’ for people registered as born in a different country than the one they were registered in (see sections ‘Migrant TB data’ and ‘Population data’). National Bureaus of Stastitics usually do not register people in process of seeking asylum and undocumented persons. National TB Registers register all TB cases, including TB in patients awaiting outcome of asylum request and undocumented migrants. We used the term ‘recently arrived migrant’ for migrants who had stayed less than 365 days in the destination country.

Countries reporting to the European Surveillance System (TESSy) use the EU TB case definition published by the European Commission [21]. Designated experts within national public health institutes of all EU/EEA countries annually submit the TB surveillance data to TESSy [3]. The current study did not address TBI, defined as a state of persistent immune response to stimulation by *Mycobacterium tuberculosis* antigens with no clinically manifested TB disease, which is not systematically collected by TESSy.

We analysed migrant TB data reported to TESSy (new, relapse, and re-treatment cases) in 2014–2020 for 20 EU/EEA countries. We calculated the average annual TB CIRs in migrants per year, destination (i.e. TB reporting) country and migrant country of origin (further: ‘origin country’). We identified origin countries with the highest numbers of migrant TB patients, as well as origin countries with the highest migrant CIRs. To better understand the risk of TB progression related to the time of immigration, the analysis was performed separately for the overall migrant population and for the subpopulation of recently arrived migrants. CIRs were compared with the WHO incidence estimates for the origin countries. Demographic characteristics of the patients were not analysed, as these were addressed in detail in an additional study performed by the authors (data not shown).

### Inclusion criteria/study population

European countries were eligible for the study if they met the low incidence country definition by having a median TB notification rate of < 10 per 100,000 population per year throughout the years 2014–2020 (27/55 European countries and territories according to WHO classification, of which 22/30 are EU/EEA countries), and if TB notification data were reported to TESSy. Liechtenstein was excluded from the study due to small observation number (two reported migrant TB patients over the study period). This led to the inclusion of 21 EU/EEA countries: Austria, Belgium, Croatia, Cyprus, Czechia, Denmark, Finland, France, Germany, Greece, Hungary, Iceland, Ireland, Italy, Luxembourg, the Netherlands, Norway, Slovakia, Slovenia, Spain, Sweden, as well as Switzerland and the UK (23 countries in total).

### Migrant TB data

Migrant TB case data for 2014–2020 were retreived from TESSy. Country of birth reported as different than reporting country was used to define migrant TB patients. Where country of birth was not available, reported nationality served as a proxy. All migrant TB patients and the corresponding migrant populations for which (i) country of birth or nationality was not reported, or reported as ‘unknown’, and/or (ii) the reported migrant population size in the given reporting country was smaller than the number of reported TB patients in that country, were excluded from the analysis. TB data were partially not available and therefore excluded from the analysis for specific years for Spain (2014), Switzerland (2019–2020) and the UK (2020), with the latter two countries reporting to the WHO rather than to TESSy on those years.

Recently arrived migrant TB patients were defined as migrants reported with TB within 365 days after arrival to the destination country. While the exact date of TB notification was available, only year of arrival (y.a.) and not the specific date was requested from TESSy, as it is reported by more countries. Therefore, we could not determine the exact length of stay in the country before diagnosis. Instead, we assumed that on average, 50% of TB patients reported in the calendar year following the reported y.a. were diagnosed within 365 days after the actual arrival. This assumption was validated against data from the Netherlands (data not shown). Thus, number of TB patients in each cohort of recently arrived migrants, defined by the same reported y.a., was estimated according to the formula:

*N* = *X* + (0.5 × *Y*)

Where:

N – number of migrant TB patients among recent migrants with a given y.a. in the destination country;

X – number of migrant TB patients reported in the same calendar year as their y.a. in the destination country;

Y – number of migrant TB patients reported in the calendar year following their y.a. in the destination country.

The y.a. variable was available in TESSy for 2017–2020. Seven countries reported the y.a. for  ≥ 70% of patients: Austria, Belgium, Czechia, Iceland, the Netherlands, Slovenia and Sweden, and were included in the CIR calculations for recently arrived migrants with TB. For comparison with a more conservative definition of recent migrants, we also present in Supplementary Table 1 the number of TB patients in migrants both arriving and being notified with TB in the same calendar year.

The number of recently arrived migrants used as denominator for calculating the CIR for those with TB in a given year, was the number of migrants with reported y.a. in the destination country in this given year (see ‘Population data’).

### Population data

Migrant population data for the included countries were extracted from Eurostat or, if not available (Croatia, Cyprus, Germany, Greece, Ireland and Switzerland) or incomplete (Luxembourg, Spain), requested from the national statistical offices. For Croatia, Cyprus and Greece data could not be acquired. Consequently, 20 countries remained in the study (Table 1).

**Table 1:** Migrant population data source, covered time period and number of reported origin countries for European destination countries included in the analysis, 2014–2020 (n = 20 destination countries)

Country	Data source	Years	Number of reported origin countries and territories^a^
Austria	Eurostat	2014–2020	229
Belgium	Eurostat	2014–2020	229
Czechia	Eurostat	2014–2020	229
Denmark	Eurostat	2014–2020	229
Finland	Eurostat	2014–2020	229
France	Eurostat	2014–2020	142
Germany	National Statistical Office^b^	2014–2020	200
Hungary	Eurostat	2014–2020	229
Iceland	Eurostat	2014–2020	229
Ireland	National Census^c^	2016	95
Italy	Eurostat	2014–2020	229
Luxembourg	Eurostat	2017–2020	228
Netherlands	Eurostat	2014–2020	229
Norway	Eurostat	2014–2020	227
Slovakia	Eurostat	2014–2020	229
Slovenia	Eurostat	2014–2020	229
Spain	National Statistical Office^d^	2014–2020	114
Sweden	Eurostat	2014–2020	229
Switzerland	National Statistical Office	2014–2018	229
United Kingdom	Eurostat	2014–2019	40

^a^ Origin countries were described as in Eurostat. Certain countries reported data on migrants from a subgroup of most common origin countries (e.g. France: 142, United Kingdom: 40).

^b^ Germany data were available in the open-access database of the Federal Statistical Office [35]. The numbers of migrants were based on the nationality of foreigners who registered with a new address in Germany, which is obligatory within 2 weeks of arrival in Germany.

^c^ Ireland delivered data from a census conducted in 2016, which was used as denominator for the entire study period 2014–2020.

^d^ Spain data were derived from the intercensal estimates of the population up to 2021.

Population data on recent migrants for 2017–2019 were extracted from Eurostat for the seven destination countries where y.a. was reported for > 70% of migrant TB patients. Eurostat records people residing in a country for a period that is, or is expected to be,  ≥ 12 months. Data for 2020 were not extracted, as the requested TESSy reporting data did not include 2021, which was a prerequisite to calculate CIR in recent migrants in our study.

### Data analysis

Annual migrant TB CIRs per 100,000 population were calculated using denominators stratified by origin country for all migrant TB patients from 2014 to 2020 for the 20 destination countries, and for recently arrived migrants for the populations that arrived in 2017–2019 in the seven destination countries. The average annual migrant CIR for each origin country was calculated and compared with the average incidence estimate published by WHO for the corresponding time period [22]. The CIRs were calculated for all available countries of origin, study years, and destination countries, and are available upon request.

R software was used to analyse the data [23].

## Results

Between 2014 and 2020, a total of 104,371 migrant TB patients were reported to TESSy by the 20 European destination countries.

### Crude incidence rates – all migrant TB patients

Germany had the largest average annual migrant population (> 10 million), and the highest average annual number of migrants with TB (Table 2). Migrant TB CIRs in the destination countries ranged from 4 per 100,000 in Slovakia to 43 per 100,000 in the UK.

**Table 2:** Average migrant population per year, average number of reported TB patients in migrants per year and average annual TB CIR in migrants to European destination countries found in the study, 2014–2020 (n = 20 destination countries^a^)

Reporting country^a^	Average migrant population per year	Average number of TB patients in migrants per year	Average annual TB CIR among all migrants (per 100,000)
Austria	1,615,659	351	22
Belgium	1,881,022	644	34
Czechia	458,469	132	29
Denmark	654,459	197	30
Finland	346,533	93	27
France^b^	8,168,326	2,889	35
Germany	10,114,912	3,673	36
Hungary	519,278	58	11
Iceland	48,965	8	16
Ireland	776,083	136	18
Italy	5,982,523	1,863	31
Luxembourg^c^	285,631	33	12
Netherlands	2,151,364	588	27
Norway	793,768	217	27
Slovakia	186,220	8	4
Slovenia	250,921	40	16
Spain	6,425,198	1,256	20
Sweden	1,777,486	508	29
Switzerland^d^	2,318,384	391	17
United Kingdom^e^	6,380,886	2,755	43

CIR: crude incidence rate; TESSy: The European Surveillance System.

^a^ Countries in the table are listed alphabetically.

^b^ For France, no migrant TB patients with specified countries of birth/nationality were reported in TESSy in 2014.

^c^ For Luxembourg, no migrant population data were available in Eurostat for 2014–2017.

^d^ For Switzerland, no TB patients were reported in TESSy in 2019 and 2020.

^e^ For the United Kingdom, no TB patients were reported in TESSy in 2020.

The average annual TB CIRs in migrants peaked in 2016 (35/100,000), followed by a slight decrease over the following years and a sharp drop in 2020 (22/100,000) as shown in Supplementary Table 2.

The highest average annual number of migrant TB patients was found in migrants from India (n = 1,366), Romania (n = 1,192), and Pakistan (n = 1,043) (Table 3A). The highest annual average CIRs were found in migrants from Eritrea (480/100,000), Somalia (414/100,000) and The Gambia (343/100,000) (Table 3B). The numbers of TB patients notified among migrants from the 20 origin countries with the highest average annual migrant TB CIRs were relatively stable over the years as illustrated in Supplementary Figure 1.

**Table 3:** Among European destination countries included in the analysis^a^, (A) top 20 origins^b^ from where the highest numbers of migrant TB patients were reported, with respective TB CIRs, and (B) top 20 origins with the highest TB CIRs in migrants, 2014–2020 (n = 33 origins listed in total)

Origin^b^	Average migrant population per year	Average number of TB patients among migrants per year	Average annual TB CIR among migrants (per 100,000)
A. Origin countries and territories sorted by highest numbers of migrant TB patients (top 20)			
India	1,233,697	1,366	111
Romania	3,131,812	1,192	38
Pakistan	836,736	1,043	125
Morocco	2,603,300	977	38
Somalia	207,822	861	414
Eritrea	154,323	740	480
Afghanistan	416,329	439	105
Senegal	307,849	344	112
Philippines	469,886	313	67
Bangladesh	389,296	274	70
Poland	2,464,274	267	11
Nigeria	403,557	265	66
Türkiye	2,351,334	264	11
Algeria	1,343,732	261	19
Guinea	99,176	257	259
China	1,038,476	220	21
The Gambia	59,830	205	343
Mali	118,966	203	171
Ethiopia	117,550	201	171
Syria	947,973	199	21
B. Origin countries and territories sorted by highest CIRs of migrant TB patients (top 20)			
Eritrea	154,323	740	480
Somalia	207,822	861	414
The Gambia	59,830	205	343
Sudan	41,163	137	333
Greenland	16,630	50	301
Chad	10,747	29	270
Guinea	99,176	257	259
Congo	87,706	168	192
Guinea-Bissau	16,203	30	185
Ethiopia	117,550	201	171
Mali	118,966	203	171
Sierra Leone	16,524	28	169
Mongolia	22,785	35	154
Nepal	33,100	49	148
Niger	11,073	16	144
Central African Republic	19,066	27	142
Djibouti	9,699	13	134
Georgia	72,422	95	131
Pakistan	836,736	1,043	125
Mauritania	32,109	36	112

CIR: crude incidence rate; TESSy: the European Surveillance System.

^a^ Destination countries included Austria, Belgium, Czechia, Denmark, Finland, France, Germany, Hungary, Iceland, Ireland, Italy, Luxembourg, Netherlands, Norway, Slovakia, Slovenia, Spain, Sweden, Switzerland and the United Kingdom. The analysis relied on data reported in TESSy.

^b^ Migrants’ origin was based on place of birth, and when this information was unavailable, on nationality.

Numbers of migrants with TB as well as TB CIRs from a given origin country varied between destination countries. For example, while 13 of 20 destination countries reported patients from Somalia, most patients from the Democratic Republic of Congo were reported by France (915 of 1,164, 78.6%, as described in Supplementary Table 3), and 49 of 50 patients from Greenland were reported by Denmark. Among the countries who reported TB patients from Eritrea, the highest TB CIR for those migrants was recorded in Belgium (2,639/100,000) and the lowest in Norway (185/100,000; Supplementary Table 3).

**Figure 1:**
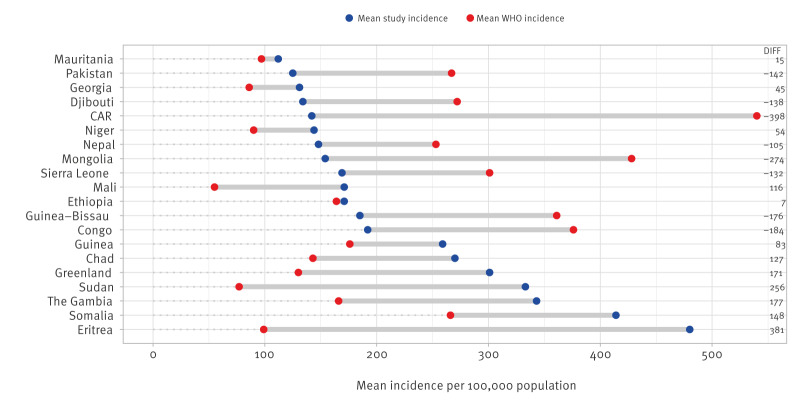
WHO-estimated average TB incidences (per 100,000 population) in 20 countries and territories^a^ compared with study-estimated average TB CIRs using data on migrants^b^ originating from these respective countries and territories^c^, 2014–2020

CAR: Central African Republic; CIR: crude incidence rate; DIFF: difference; WHO: World Health Organization.

^a^ The countries and territories depicted represent the 20 migrant origins found in the study to have the highest TB CIRs.

^b^ Migrants’ destination countries included Austria, Belgium, Czechia, Denmark, Finland, France, Germany, Hungary, Iceland, Ireland, Italy, Luxembourg, Netherlands, Norway, Slovakia, Slovenia, Spain, Sweden and Switzerland.

^c^ Migrants’ origin was based on place of birth, and when this information was unavailable, on nationality.

For 10 of the 20 origin countries with highest CIRs among migrants, the study CIRs were substantially higher than the WHO incidence estimates for the corresponding time period [22] (difference in incidence > 20/100,000), and for eight of the 20 countries they were substantially lower (Figure 1). For Ethiopia and Mauritania, the numbers corresponded. Large differences (> 200/100,000) were seen in Eritrea and Sudan, where the CIRs among migrants were higher, and in Central African Republic (CAR) and Mongolia, where the WHO estimates were higher.

On the left Y axis, countries are sorted from top to bottom according to highest to lowest study-estimated CIR. On the right Y axis, differences between the study mean TB CIR and the WHO mean TB incidence for each country are shown.

### Crude incidence rates – recently arrived migrant TB patients

In each destination country, the CIRs in recent migrants (Table 4) appeared about two times higher than compared with all migrants (Table 2) and covered a higher range between the destination countries with lowest and highest CIR (26–84/100,000 for recent migrants vs 4–43/100,000 for all migrants).

**Table 4:** Destination-country specific average TB CIRs in recently arrived migrants^a^ 2017–2019; average TB CIRs in all migrants 2014–2020^b^ are shown for comparison (n = 7 destination countries)

Destination (reporting) country	Average annual number in 2017–2019 of:		Average annual TB CIR (per 100,000) in	
	Recently arrived migrants^a^	TB patients in recently arrived migrants^a^	Recently arrived migrants 2017–2019	All migrants^b^ 2014–2020
Austria	101,105	62	61	22
Belgium	123,135	103	84	34
Czechia	66,508	31	47	29
Iceland	9,602	4	42	16
Netherlands	170,279	121	71	27
Slovenia	23,437	6	26	16
Sweden	117,903	67	56	29

CIR: crude incidence rate.

^a^ TB patients in recently arrived migrants are defined as having been diagnosed with TB within 1 year after arrival in the destination country. Recently arrived migrants are migrants that arrived in their destination country in the given year considered within the period analysed.

^b^ These data are from Table 2.

Variation in CIRs among the 20 origin countries of recently arrived migrants with TB with most patients reported was substantial (Table 5A), and higher compared with total migrants for every listed country except India, Afghanistan, and Syria where the CIR were comparable in all migrants and recent migrants (Table 3A).

**Table 5:** Among destination countries included in the analysis^a^ (A) top 20 origins^b^ from where the highest numbers of TB patients in recent migrants^c^ were reported, with respective TB CIRs, and (B) top 20 origins with the highest TB CIRs in recent migrants 2017–2019; TB CIRs in all migrants 2014–2020 are shown for comparison (n = 24 origins)

Origin^b^	Average annual number in 2017–2019 of		Average annual TB CIR (per 100,000) in	
	Recently arrived migrants^c^	TB patients in recently arrived migrants^c^	Recently arrived migrants^c^ 2017–2019	All migrants^a^ 2014–2020
A. Origin countries sorted by highest numbers of recent migrant TB patients (top 20)				
Eritrea	6,820	76	1,112	480
Somalia	4,708	29	605	414
Romania	35,367	28	80	38
India	21,168	22	102	111
Ukraine	25,711	15	58	19
Afghanistan	12,930	14	104	105
Ethiopia	3,537	12	339	171
Morocco	10,692	10	94	38
Georgia	1,355	10	713	131
Indonesia	2,314	10	418	59
Philippines	3,952	9	215	67
DRC	2,821	8	266	51
Guinea	1,649	8	455	259
Syria	30,626	7	23	21
Poland	34,643	7	20	11
China	13,073	7	51	21
Sudan	1,284	7	506	333
Bulgaria	15,808	6	39	19
The Gambia	548	5	974	343
Mongolia	2,389	5	216	154
B. Origin countries sorted by highest CIRs of migrant TB patients (top 20)				
Eritrea	6,820	76	1,112	480
The Gambia	548	5	974	343
Georgia	1,355	10	713	131
Somalia	4,708	29	605	414
Sudan	1,284	7	506	333
Guinea	1,649	8	455	259
Indonesia	2,314	10	418	59
Ethiopia	3,537	12	339	171
DRC	2,821	8	266	51
Mongolia	2,389	5	216	154
Philippines	3,952	9	215	67
Nigeria	2,360	4	184	66
Thailand	3,724	5	125	48
Afghanistan	12,930	14	104	105
India	21,168	22	102	111
Morocco	10,692	10	94	38
Vietnam	5,098	4	85	48
Romania	35,367	28	80	38
Ukraine	25,711	15	58	19
China	13,073	7	51	21

DRC: Democratic Republic of the Congo.

^a^ For recent migrants and recent migrants with TB (2017–2019), destination countries included in the analysis were Austria, Belgium, Czechia, Iceland, the Netherlands, Slovenia and Sweden; for all migrants and migrants with TB (2014–2020), destination countries in the analysis additionally included Denmark, Finland, France, Germany, Hungary, Ireland, Italy, Luxembourg, Norway, Slovakia, Spain, Switzerland and the United Kingdom. The analysis relied on data reported to TESSy.

^b^ Only origins with an average of  ≥ 4 reported patients per year are shown. Migrants’ origin was based on place of birth, and when this information was unavailable, on nationality.

^c^ TB patients in recently arrived migrants are defined as having been diagnosed with TB within 1 year after arrival in the destination country. Recently arrived migrants are migrants who arrived in their destination country in the year considered within the period analysed.

For the 17 countries with highest CIRs (except India, Afghanistan and Syria), the CIRs among recent migrants were more than 1.4 times as high as the overall migrant CIR for the same country (Table 3B, Table 5B).

The calculation of CIR based on the more conservative definition of recent migrants with TB (as those who were reported in the same calendar year as they entered the destination country) resulted in lower numbers of TB patients and lower CIRs, but the relative magnitude of number of TB patients and incidences between the origin countries remained largely unchanged, as described in Supplementary Table 4.

**Figure 2:**
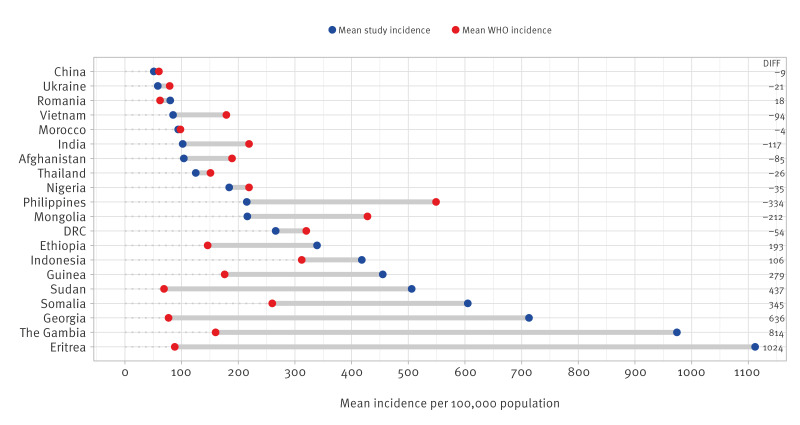
WHO-estimated average TB incidence (per 100,000 population) for 20 countries^a^ compared with study-estimated average TB CIRs using data on recently^b^ arrived migrants^c^ originating from these respective countries^d^, 2017–2019

CIR: crude incidence rate; DRC: Democratic Republic of the Congo; DIFF: difference; WHO: World Health Organization.

^a^ The countries depicted represent the 20 migrant origins found in the study to have the highest TB CIRs.

^b^ Recently arrived migrants are migrants who arrived in their destination country in the year for which the annual CIR was calculated. TB patients in recently arrived migrants are defined as having been diagnosed with TB within 1 year after arrival in the destination country.

^c^ Migrants’ destination countries included Austria, Belgium, Czechia, Iceland, the Netherlands, Slovenia and Sweden.

^d^ Migrants’ origin was based on place of birth, and when this information was unavailable, on nationality.

Figure 2 shows the difference between the 20 origin countries with the highest TB CIRs in recent migrants and the WHO estimated incidence for those countries [22]. For eight of the 20 countries the study CIR was substantially higher (difference in incidence > 20/100,000), and for nine of the 20 countries the study CIR was substantially lower than the WHO incidence estimate. The two numbers corresponded for three of the countries. Similar to all migrants, the largest differences (> 200/100,000) were seen in Eritrea, The Gambia, Georgia, Guinea, Somalia and Sudan where the study CIRs were higher, and in Mongolia and Philippines where the WHO estimates were higher.

On the left Y axis, countries are sorted from top to bottom according to highest to lowest study-estimated CIR. On the right Y axis, differences between the study mean TB CIR and the WHO mean TB incidence for each country are shown.

## Discussion

Our study included more than 100,000 migrant TB patients in 20 low-incidence European countries in 2014–2020. The annual CIR for all migrants varied among study years: from 35 per 100,000 in 2016 to 22 per 100,000 in 2020, and between the studied countries: from 4 per 100,000 in Slovakia to 43 per 100,000 in the UK. The CIRs in recently arrived migrants seemed higher compared with all migrants, with highest rates observed in migrants from Eritrea (1,112/100,000), The Gambia (974/100,000), and Georgia (713/100,000). The CIRs for all migrants and for recently arrived migrants from Eritrea, The Gambia and Sudan were more than twice as high as the WHO TB incidence estimates for these countries.

Migration is driven by a combination of factors, such as fleeing war, fear of persecution or natural disasters in home countries, and better economic and social opportunities in destination countries [24]. Geographic distance, historical ties, common language, political situation, all influence the size and type of migrant populations in destination countries [20]. In 2014–2020, in the destination countries included in our study, on average 13% of the total population was of migrant origin, varying from 3% in Slovakia to 46% in Luxembourg [18]. The countries of origin of migrants differed considerably, e.g. most migrants in Austria and Switzerland were from Germany, in Belgium and Spain from Morocco, in Finland from Eeastern European countries, and in the Netherlands from Turkey [18].

We analysed and presented TB among migrants in two different ways: (i) in numbers, which is highly dependent on the size of the migrant population, and (ii) as CIR, representing the risk of TB.

In 2014–2020, on average 31% of TB patients in all EU/EEA countries were migrants, while in the 20 low-incidence countries analysed in our study it was 55%, varying from 3% in Slovakia to 89% in Norway and Sweden (calculated based on the data published in the annual European Centre for Disease Prevention and Control (ECDC) reports ‘Tuberculosis Surveillance and Monitoring in Europe’ for the given years [25]). TB CIRs in the native population was on average 3.1 per 100,000, and varied from 0.6 per 100,000 in Iceland and Norway to 7.1 per 100,000 in Hungary [25]. The TB CIRs among migrants depends on the composition of the migrant population and may be very low in countries such as Slovakia where 47% of migrants originate from neighbouring Czechia, and Slovenia where 80% of migrants come from Bosnia and Herzegovina, Croatia, North Macedonia, Montenegro, Serbia, Kosovo‡ [18]. The data on the number and proportion of migrant TB patients are published annually in the ECDC/WHO/Europe TB Surveillance Report, but the report does not give information on country of origin or rates in specific migrant populations.

The eight most common countries of origin of TB patients in our study (India, Romania, Pakistan, Morocco, Somalia, Eritrea, Afghanistan, and Senegal) were the same as found by Vasiliu et al., who studied TB in migrants for 2020 in the EU/EEA, Switzerland, and the UK [20].

Besides Asian and African countries, also two EU countries, Romania and Poland, were present among 20 origin countries contributing highest numbers of migrant TB patients. The TB CIRs among migrants from those countries seemed lower than the WHO estimates for the population of those countries, as the mean TB incidence for Poland in 2014–2020 based on the WHO Global TB Reports [22] was 16 per 100,000 (11/100,000 in our study) and for Romania 70 per 100,000 (38/100,000 in our study) as presented in Supplementary Table 5. There are large migrant populations from both of those countries living in the studied destination countries (3.1 and 2.5 million respectively).

In our study, 15 of 20 highest TB CIRs were found in migrants from African countries, all with CIRs above 100 per 100,000. As a unique population, the highest TB incidence in migrants from a different continent was detected for Greenland, an autonomous territory of Denmark with a CIR of 301 per 100,000, wherefrom all patients except one were reported by Denmark.

The TB risk in migrants in low-incidence countries is determined by several factors, such as (i) the risk of being infected with *Mycobacterium tuberculosis* in the country of origin (the WHO-estimated TB incidence for countries is a proxy for this risk), (ii) exposure to *Mycobacterium tuberculosis* during the route of migration to the destination country, (iii) the risk of re-activation of TBI to TB disease in the destination country, which diminishes over time, and *(*iv) the risk of exposure in the destination country or during travel to high-incidence settings after residence [24]. The general risk of TBI and TB also depends on factors such as sex, age, and largely on socioeconomic background [26]. People fleeing war, poverty or crisis are more likely to be exposed to harsh conditions during migration and upon arrival with less or no access to healthcare, which leads to a higher risk of TB in these populations [11]. This likely explains why we found that the TB CIRs were over two times higher than WHO estimates for countries such as Eritrea, The Gambia and Sudan. On the other hand, TB CIRs in migrants from Pakistan (125/100,000), or recent migrants from India (111/100,000) and Vietnam (85/100,000), were substantially lower than the mean incidence estimates by WHO for these countries for the corresponding populations (265/100,000, 235/100,000, and 179/100,000, respectively; Figure 1 and Figure 2). This can be explained by lower risk in certain groups of recent migrants, e.g. in highly skilled labourers, and/or reduced risk of non-recent migrants due to a longer stay in the destination country [24].

In general, people who have stayed in a low-incidence destination country for a long time, have a lower risk of developing TB [27,28] compared with recent migrants. This corresponds to our study findings, where TB CIRs in recent migrants were higher in all except three countries than in all migrants from the corresponding country. We identified five African countries (Eritrea, The Gambia, Guinea, Somalia, Sudan), where TB CIRs exceeded 500 per 100,000 in recent migrants, being two to five times higher than the WHO estimates. This is likely related to the poor conditions during migration, as well as a high proportion of migrants in vulnerable situations, whose risk for TB was higher [29]. On the other hand, the TB CIR in recent migrants from the Philippines was less than 50% of the WHO estimate for this country (> 500/100,000), but still  > 200 per 100,000. Those migrants could potentially have had a lower TB risk already before migration, for example if they had a better socioeconomic background, and arrived in Europe looking for family reunification or better labour perspectives [30]. The higher risk of TB among recent migrants is an important factor for planning of TB screening programmes, and it also needs to be considered in the regular healthcare systems.

We assume that there are genuine differences between TB incidences in a given country of origin and among migrants from this country. This can be explained by the fact that the migrating population has a different TB risk level already before or changed by migration. Besides that, a different basis for calculation of TB incidence could impact the described differences: while we analysed actively acquired migrant TB and population data recorded by European low-incidence countries, the WHO estimations include modelling approaches and rely on the quality of national TB surveillance which differs between countries. Active case finding via migrant screening could also contribute to the observed differences between the CIRs calculated in the study compared with WHO incidence estimates for the origin countries.

Another way to assess TB risk in recent migrants would be in evaluating the yield of screening programmes. This has been difficult at European level due to different screening approaches of countries [31]. The E-DETECT TB, a European multi-country collaboration, developed a European database and performed cross-country pooled and comparative analyses of screening coverage, results and linkage to care in Italy, the Netherlands, Sweden, and the UK [32]. The authors however did not specify the yield per country of origin. A Swedish study assessed the TBI screening programme in Stockholm 2015–2018 and found Interferon Gamma Release Assay (IGRA) positivity in 25% of screened migrants [33], with an increased trend in IGRA-positivity observed for increased age and higher TB incidence in country of origin. Several TBI migrant screening studies were conducted in the Netherlands for newly arrived migrants, asylum seekers, and Eritrean migrants who stayed less than 5 years in the Netherlands, indicating that tailored approaches facilitate uptake of screening and high treatment completion rates [34].

Our study’s limitations are mostly related to the difficulties in acquiring a homogenous and complete dataset for all European destination countries, and to the fact that it is not feasible to record dynamic migrating populations in a way fully reflecting reality (e.g. correctly capturing undocumented migrants, migrants who change their migration status, travel to further countries or acquire new citizenship). Undocumented migrants are at least partially not captured in population statistics, whereas undocumented migrants with TB are notified in the national surveillance systems. This likely causes a systematic overestimation of the calculated CIRs. The denominator data used to calculate the CIRs have been acquired from several national sources as not all countries report to Eurostat; Eurostat data were incomplete for certain countries, e.g. UK, France and Spain. We encountered additional inconsistencies also within TESSy dataset, as for example Danish data included 2^nd^ generation migrants with TB (people born in Denmark or with Danish citizenship, whose parents were born abroad and do not possess Danish citizenship), while for the other countries only 1^st^ generation migrants (people who were born abroad or have nationality other than the destination country) were included. Reporting to Eurostat by every country and following the exact procedures of data submission for both Eurostat and TESSy would facilitate further analyses on the topic of TB and in other areas of health-related research. Besides that, TB screening policies and the time of performed screening vary considerably between countries [14]. This may have led to some bias in our data, since pre-entry screening (in the UK) may lead to fewer incident migrant TB cases in the country of destination, and screening for TB disease at time of entry may lead to earlier diagnosis of cases and a higher proportion of TB in recent migrants, which otherwise would have been diagnosed later. Knowing that TB incidence in a population of a given origin can differ between destination countries (e.g. Somalian population in Sweden was characterised with considerably higher TB incidence than the Somalian population in Italy [20]), another limitation of our study is that the selection of destination countries for the ‘all migrants’ and ‘recent migrants’ analysis was different due to data availability, which might also influence the comparison of the calculated results. To account for and minimise incoherencies and particularities arising in the complex and versatile collection of datasets used in this study, we selected a 7-year-long study period and approached the data curation particularly meticulously, including consultations with institutions and national and international surveillance experts responsible for data collection. This analysis can be used to tailor public health interventions to those most at risk and to address additional risk factors for TB like hazardous routes and conditions of migration experienced by the refugees as well as demographic and socioeconomic factors distinguishing migrants from the general populations of their origin countries. In a separate study, we describe demographic characteristics of migrants with TB in the EU/EEA low-incidence countries to better inform the prevention and control strategies (data not shown).

## Conclusion

Our study reveals considerably higher CIRs in recently arrived migrants and high CIRs in migrants from certain countries compared with the total migrant population as well as to WHO country estimates. Although these findings present challenges for a harmonisation of TB screening policies, countries could aim at more targeted approaches, based on potential common criteria which also take the country-specific migrational TB epidemiology in the destination country into account. Consistent reporting to ECDC (numerator) and Eurostat (denominator) as well as standardised and continued data monitoring and evaluation is required to establish the necessary evidence for tailored and regularly need-adapted TB prevention and control strategies.

## ‡Note

This designation is without prejudice to positions on status and is in line with United Nations Security Council Resolution 1244/99 and the International Court of Justice Opinion on the Kosovo Declaration of Independence.

## Supplementary Data

263000 Supplement S1
