# Supplementary material for: Tuberculosis rates in migrants in low-incidence European countries, according to country of origin, reporting country and recency of immigration, 2014 to 2020
Source: Euro Surveill. 2025 Mar 20;30(11):2400489. doi: 10.2807/1560-7917.ES.2025.30.11.2400489 (PMC11927070; doi:10.2807/1560-7917.ES.2025.30.11.2400489)
Supplement: Supplement S1 [file 2400489_SupplementaryMaterial.pdf]

This supplementary material is hosted by *Eurosurveillance* as supporting information alongside the article "Tuberculosis rates in migrants in low-incidence European countries, according to country of origin, reporting country and recency of immigration, 2014 to 2020", on behalf of the authors, who remain responsible for the accuracy and appropriateness of the content. The same standards for ethics, copyright, attributions and permissions as for the article apply. Supplements are not edited by *Eurosurveillance* and the journal is not responsible for the maintenance of any links or email addresses provided therein.

Supplementary tables and figures

Supplementary Table 1: Destination-country specific TB incidence rates in recently arrived migrants in seven destination countries, 2017-2019. Average annual number of TB patients and incidence in migrants both arriving and notified with TB in the given calendar year of arrival is presented.

Reporting country	Average recently arrived migrant population per year 2017-2019	Average annual number of TB patients in recently arrived migrants 2017-2019	Average annual TB incidence among recently arrived migrants 2017-2019 (per 100,000)
Austria	101 105	52	51
Belgium	123 135	75	61
Czechia	66 508	24	36
Iceland	9 602	4	38
Netherlands	170 279	88	51
Slovenia	23 437	4	18
Sweden	117 903	45	38

Supplementary Table 2: Number of migrant TB patients and number of migrants reported in EU/EEA in 2014-2020 and calculated annual TB incidence.

**For France, no migrant TB patients with specified countries of birth/nationality were reported in TESSy in 2014. For Luxembourg, no migrant population data was available in Eurostat for 2014-2017. For Switzerland, no TB patients were reported in TESSy in 2019 and 2020. For UK, no TB patients were reported in TESSy in 2020.*

Year	Number of TB patients among migrants	Migrant population	TB incidence among migrants (per 100,000)
2014*	12 303	38 399 176	32
2015*	16 379	47 871 607	34
2016*	17 139	49 621 936	35
2017*	16 885	51 651 912	33
2018	16 444	52 926 164	31
2019*	14 942	51 410 410	29
2020*	10 279	46 028 548	22

Supplementary Table 3: Distribution of migrants and migrant TB patients from Somalia, Eritrea and Democratic Republic of the Congo among the destination countries.

Reporting country	Average number of TB patients among migrants per year	Average migrant population per year	Average annual TB incidence among migrants (per 100,000)
**A. Country of birth: Democratic Republic of the Congo**			
Austria	1	314	318
Belgium	9	3 981	226
Czechia	0	61	0
Denmark	1	498	201
Finland	0	121	0
France	131	69 999	187
Germany	7	1 676	418
Hungary	0	79	0
Iceland	0	3	0
Ireland	2	2 722	73
Italy	8	3 169	252
Luxembourg	0	165	0
Netherlands	2	931	215
Norway	0	259	0
Slovakia	0	24	0
Slovenia	0	7	0
Spain	2	2 011	99
Sweden	5	831	602
Switzerland	0	855	0
**B. Country of birth: Eritrea**			
Austria	1	290	345
Belgium	19	720	2 639
Czechia	0	9	0
Denmark	16	3 598	445
Finland	3	459	654
France	16	1 603	998
Germany	374	59 100	633
Hungary	0	35	0
Iceland	0	9	0
Ireland	1	164	610
Italy	29	12 438	233
Luxembourg	5	327	1 529
Netherlands	93	10 216	910
Norway	35	18 967	185
Slovenia	0	27	0
Sweden	86	32 756	263
Switzerland	62	13 604	456
**C. Country of birth: Somalia**			

Austria	49	4 573	1 072
Belgium	30	5 671	529
Czechia	0	25	0
Denmark	20	11 167	179
Finland	23	10 955	210
France	26	4 015	648
Germany	381	35 380	1 077
Hungary	0	582	0
Iceland	1	17	5 882
Ireland	3	1 500	200
Italy	59	12 034	490
Luxembourg	0	47	0
Netherlands	63	26 076	242
Norway	39	28 054	139
Slovakia	0	15	0
Slovenia	0	27	0
Sweden	135	63 118	214
Switzerland	32	4 566	701

Supplementary Table 4: The top 20 origin countries for recent migrants reported in TESSy in 2017-2019, with A. highest numbers of TB patients, B. highest TB crude incidence rates (CIR) among migrants. Average annual number of TB patients and incidence in recently arrived migrants based on number of migrants notified with TB in the given calendar year of arrival is presented. DRC – Democratic Republic of the Congo

**Country of birth**	**Average annual number of TB patients in recently arrived migrants 2017-2019**	**Average population recently arrived migrants per year 2017-2019**	**Average annual TB CIR in recently arrived migrants 2017-2019 (per 100,000)**
A. Origin countries sorted by highest numbers of migrant TB patients (top 20)			
Eritrea	56	6 820	826
Romania	23	35 367	65
Somalia	20	4 708	432
Ukraine	14	25 711	53
India	12	21 168	58
Georgia	9	1 355	639
Afghanistan	8	12 930	64
Ethiopia	7	3 537	207
Morocco	7	10 692	62
Indonesia	7	2 314	302
DRC	6	2 821	225
Guinea	6	1 649	364
China	6	13 073	48
Philippines	5	3 952	135
Syria	5	30 626	15
Poland	5	34 643	15
Sudan	5	1 284	389
Bulgaria	5	15 808	30

The Gambia	4	548	730
Mongolia	4	2 389	181
B. Origin countries sorted by highest CIRs of migrant TB patients (top 20)			
Eritrea	56	6 820	826
The Gambia	4	548	730
Georgia	9	1 355	639
Somalia	20	4 708	432
Sudan	5	1 284	389
Guinea	6	1 649	364
Indonesia	7	2 314	302
DRC	6	2 821	225
Ethiopia	7	3 537	207
Mongolia	4	2 389	181
Nigeria	4	2 360	170
Philippines	5	3 952	135
Thailand	4	3 724	98
Romania	23	35 367	65
Afghanistan	8	12 930	64
Morocco	7	10 692	62
Vietnam	3	5 098	59
India	28	21 168	80
Ukraine	15	25 711	58
China	7	13 073	51

Supplementary Table 5: TB incidence estimates for Poland and Romania 2014-2020 by WHO, as reported in Global Tuberculosis Reports for given years, mean TB incidence 2014-2020 based on the WHO data, and mean crude incidence rate (CIR) of TB among migrants from Poland and Romania calculated in the study.

country	WHO incidence estimate 2014	WHO incidence estimate 2015	WHO incidence estimate 2016	WHO incidence estimate 2017	WHO incidence estimate 2018	WHO incidence estimate 2019	WHO incidence estimate 2020	Mean TB incidence 2014-2020 based on WHO estimates	Calculated mean CIR among migrants, 2014-2020
Poland	19	19	18	17	16	15	9	16	11
Romania	85	82	74	72	68	65	43	70	38

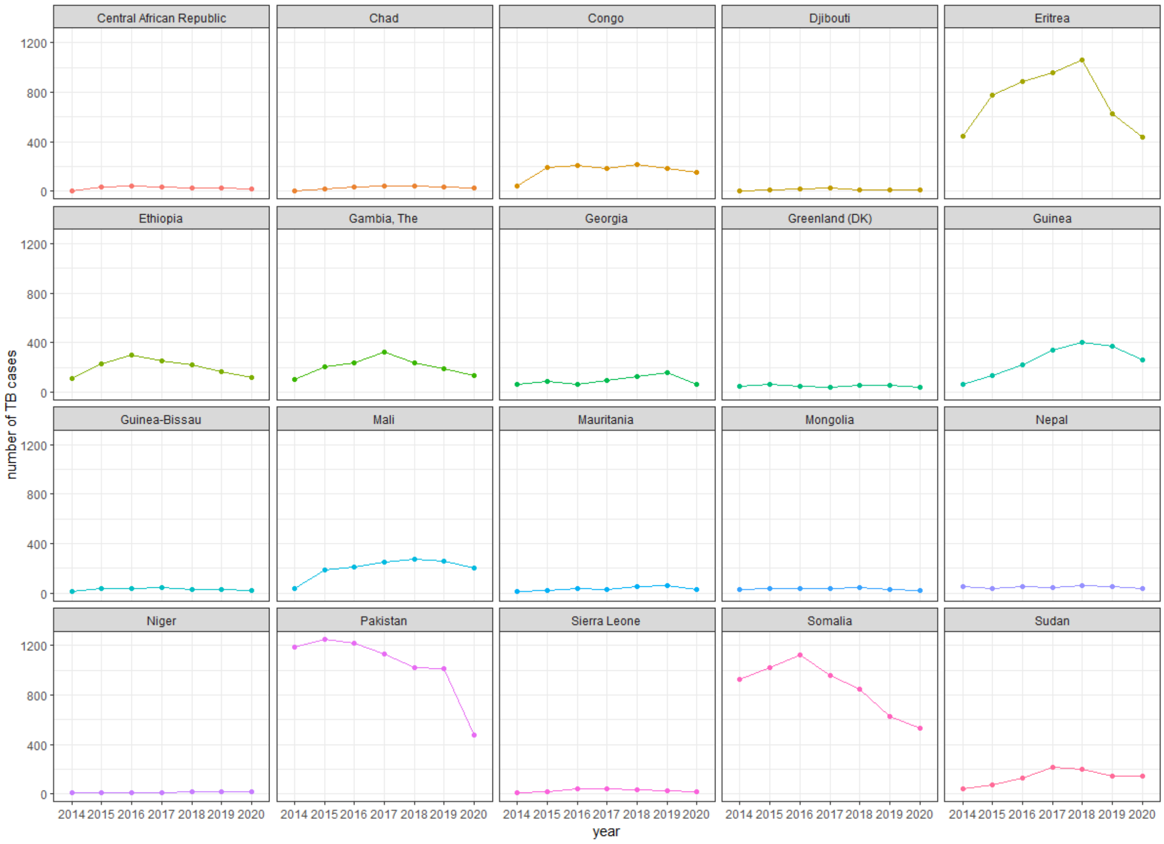

Supplementary Figure 1: Number of TB patients reported in EU/EEA from the 20 countries of origin presenting highest reported CIR in EU/EEA, from which at least 70 TB patients have been reported in TESSy in 2014-2020 in the EU/EEA destination countries included in the analysis.
